# Meta-analysis of the correlation between selenium and incidence of hepatocellular carcinoma

**DOI:** 10.18632/oncotarget.12804

**Published:** 2016-10-21

**Authors:** Ziwei Zhang, Mingyu Bi, Qi Liu, Jie Yang, Shiwen Xu

**Affiliations:** ^1^ College of Veterinary Medicine, Northeast Agricultural University, Harbin 150030, China; ^2^ Harbin Railway Public Security Bureau Police Dog Base, Harbin 150056, P. R. China

**Keywords:** hepatocellular carcinoma, selenium, correlation, meta-analysis

## Abstract

Hepatocellular carcinoma (HCC) is the most common cancer type. There is a correlation between selenium (Se) deficiency and the incidence of HCC. To clarify the effects of Se level on the risk of HCC patients, a meta-analysis was performed. A total of 9 articles published between 1994 and 2016 worldwide were selected through searching PubMed, EMBASE, web of science, Cochrane Library, Springer Link, Chinese National Knowledge Infrastructure (CNKI), and Chinese Biology Medicine (CBM), and the information were analyzed using a meta-analysis method. Heterogeneity was assessed by using the I^2^ index. Publication bias was evaluated by Begg's Test analysis. Pooled analysis indicated that patients with HCC had lower Se levels than the healthy controls [standardized mean difference (SMD)= −1.08, 95% confidence intercal (CI) = (−0.136, −0.08), *P* < 0.001]. Further subgroup analysis showed this effect to be independent of the study design, race or sample collection. In conclusion, this meta-analysis suggested an inverse correlation between Se level and the risk of HCC in humans patients.

## INTRODUCTION

Hepatocellular carcinoma (HCC) is the most common liver cancer worldwide. HCC is as a result of either metabolic toxins such as alcohol or aflatoxin, a viral hepatitis infection (hepatitis B or C) [1–4]. Furthermore, it has been reported that oxidative stress is a common inducement of liver diseases that excessive reactive oxygen (ROS) in body cause mutations in cancer [5, 6].

Micronutrients may reduce the risk of cancer, among which selenium (Se) is of particular interest [7, 8]. Se is an essential micronutrient required for human health and has been studied for its antioxidant and anticancer properties, specifically against HCC. Se is thought to protect macromolecules and membrane lipids from oxidative damage by combating ROS [9, 10]. The following articles affirm the relationship between Se concentration and HCC. A matched-case-control study revealed lower blood Se levels among HCC patients [11]. There is another report on the obvious correlation relationship between low Se level and HCC in Korean hepatoma patients [12]. In contrast, the following article denies the relationship between Se in liver tissues and liver cancer. No relationship was observed Se concentration and HCC incidence [13].

Several studies have investigated the relationship between Se level and HCC risk [11–21]. Yu et al. [19, 22] found that there was asignificant inverse correlation between the levels of blood Se and HCC risk, while others have not demonstrated this [13]. Thus, we conduct a systematic review on the relationship between Se level and HCC risk.

## RESULTS

### Eligible studies

**Figure 1:**
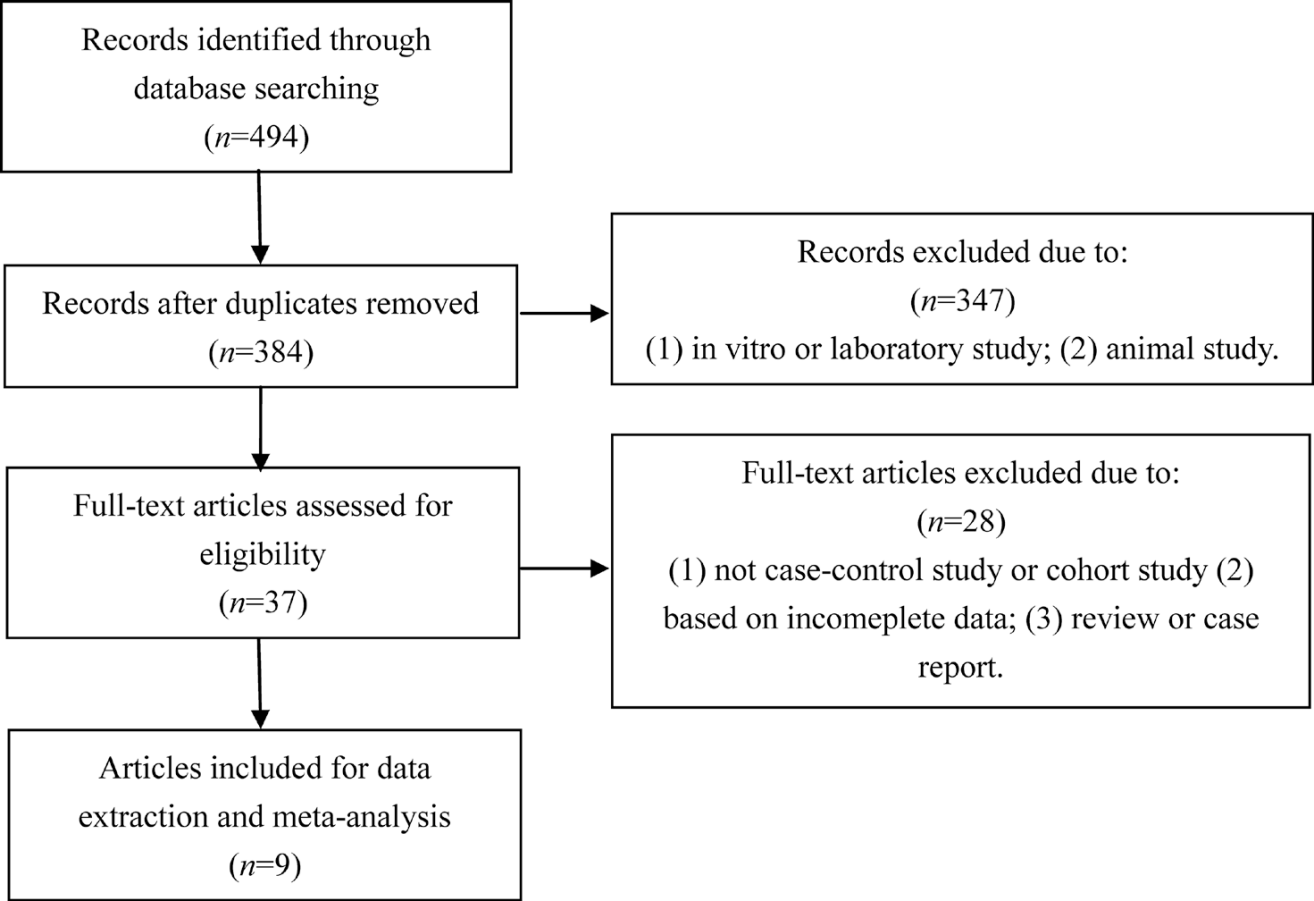
Flow chart of the study

With the search strategy stated before, 9 relevant records were included in our meta-analysis and data were extracted (Figure 1) [11, 12, 16, 17, 19–21, 23, 24]. Table 1 summarized the characteristics of the 9 enrolled studies. There were 6 case-control studies and 3 cohort records. Six studies were conducted among Asian and 3 among Caucasian. In 3 studies, Se status was based on analysis of serum, whereas in the remaining 5 studies, blood was the sample specimen used and in 1 study, toenail Se status was used. 525 cases and 908 control subjects were enrolled in our meta-analysis.

**Table 1:** Baseline characteristics of included trials

First author	Year	Study design	Race	Age	Female	Male	Follow-up	Collection	Case	Control	Selenium concentration	
											Case	Control
Massimo Casaril	1994	case-control	Caucasian	21–74	6	17	NA	Serum	23	19	78.35 ± 19.78 ng/ml	96.09 ± 22.03 ng/ml
TE-HSIEN LIN	1998	case-control	Asian	21–82	NA	NA	NA	blood	51	19	106 ± 17.7 μg/l	126.4 ± 10.1 μg/l
Ming-Whei Yu	1999	cohort	Asian	30–65	NA	NA	4	blood	69	138	131.6 ± 30.9 μg/l	150.2 ± 35.2 μg/l
Wang Chin-Thin1	2002	case-control	Asian	42–69	NA	51	2	blood	51	50	0.18 ± 0.02 μg/ml	0.28 ± 0.06 μg/ml
CHING-CHIANG Lin	2006	case-control	Asian	35–61	9	9	NA	Serum	18	50	108.5 ± 21.8 μg/l	129 ± 21.5 μg/l
In-Wook Kim	2012	case-control	Asian	40–59	7	23	NA	Serum	30	120	67.47 ± 14.3 μg/l	108.38 ± 29.5 μg/l
Dominik Bettinger	2013	case-control	Caucasian	NA	NA	10	NA	blood	10	10	85 ± 11.5 μg/l	117.5 ± 15.7 μg/l
David J Hughes	2016	cohort	Caucasian	25–70	NA	NA	4	blood	107	108	74.127 ± 19.29 μg/l	87.309 ± 18.582 μg/l
Lori C.Sakoda	2005	cohort	Asian	NA	12	154	8	toenial	166	394	3.1 ± 0.333 ppm	3.5 ± 0.45 ppm

NA, not available.

### Quantitative synthesis

**Figure 2:**
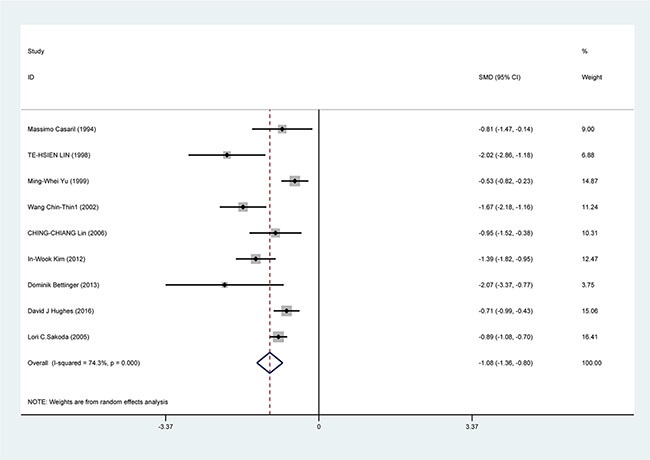
Forest plots for meta-analysis of the correlation of Se level with HCC risk Square represents effect estimate of individual studies with their 95% confidence intervals. In this chart, studies are stored in order of years of publication and author's names, based on a random effects model.

The result of random-effects meta-analysis showed that Se levels was inversely correlated with HCC [standardized mean difference (SMD) = −1.08, 95% CI = (−0.136, −0.08), *P* < 0.001]. The result of pool analysis showed lower Se level had a relationship with HCC with obvious heterogeneity (I^2^ = 74.3%, *P* < 0.001) (Figure 2).

**Figure 3:**
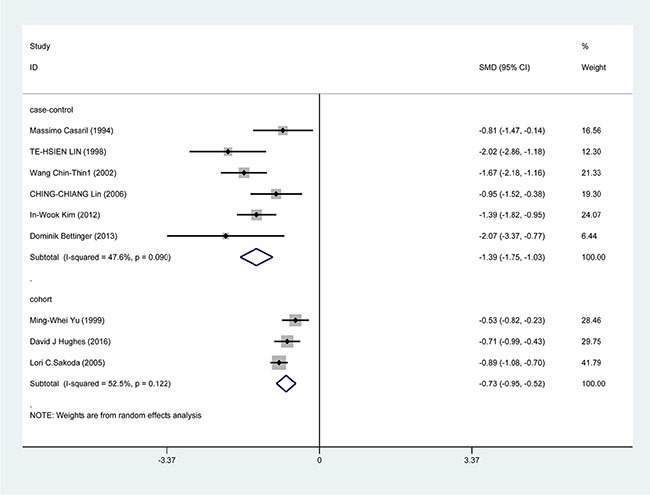
Forest plots for meta-analysis of in subgroups by study design in the correlation of Se level in with HCC risk Square represents effect estimate of individual studies with their 95% confidence intervals. In this chart, studies are stored in order of years of publication and author's names, based on a random effects model..

**Figure 4:**
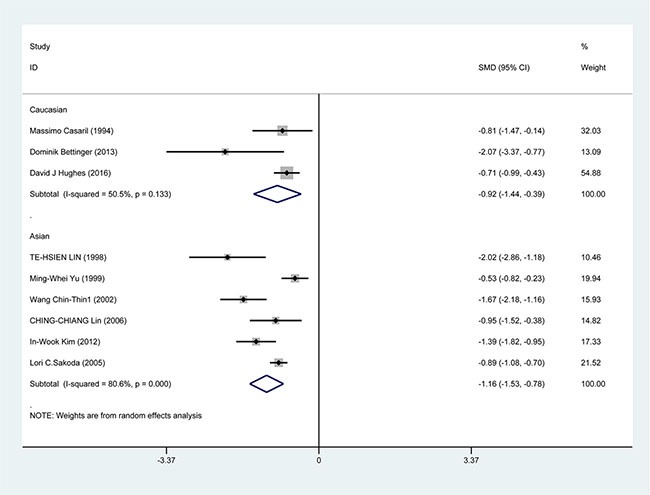
Forest plots for meta-analysis of in subgroups by race in the correlation of Se level in with HCC risk Square represents effect estimate of individual studies with their 95% confidence intervals. In this chart, studies are stored in order of years of publication and author's names, based on a random effects model.

**Figure 5:**
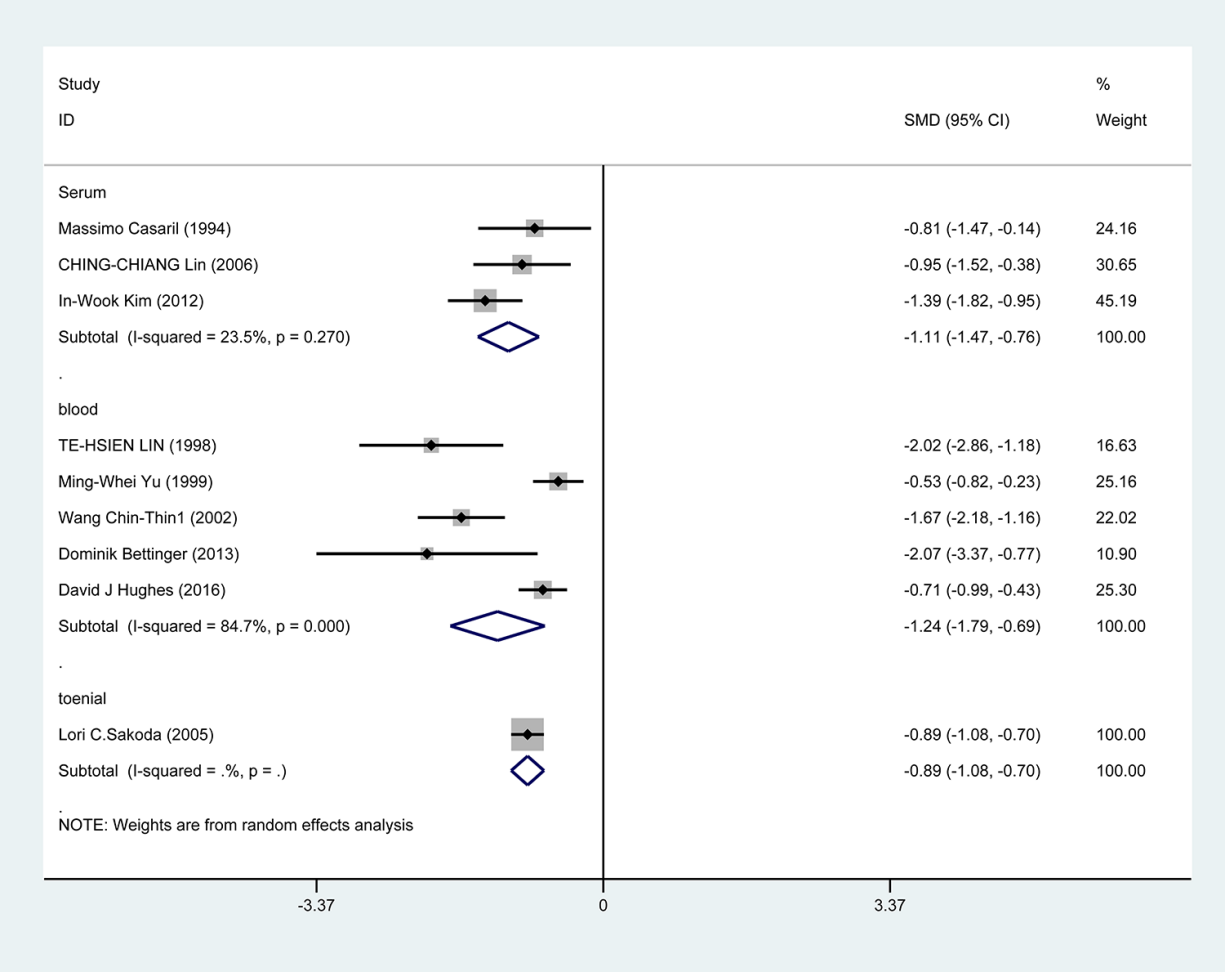
Forest plots for meta-analysis of in subgroups by collection in the correlation of Se level in with HCC risk Square represents effect estimate of individual studies with their 95% confidence intervals. In this chart, studies are stored in order of years of publication and author's names, based on a random effects model.

In the subgroup analysis by study design, a significant correlation was found in case-control study [SMD = −1.388, 95% CI = (−1.751, −1.026), *P* < 0.001] and cohort study [SMD = −0.733, 95% CI = (−0.945, −0.520), *P* < 0.001] (Figure 3). In the race subgroup analysis, lower Se level was also found in Caucasian [SMD = −0.918, 95% CI = (−1.443, −0.393), *P* = 0.001] and in Asian (SMD = −1.115, 95% CI = (−1.526, −0.784), *P* < 0.001] (Figure 4). In the subgroup analysis by collection, the inverse correlation between Se level and HCC were observed in serum [SMD = −1.113, 95% CI = (−1.472, −0.755), *P* < 0.001], blood [SMD = −1.241, 95% CI = (−1.795, −0.678), *P* < 0.001] and toenail [SMD = −0.889, 95% CI = (−1.081, −0.697), *P* < 0.001] (Figure 5).

Publication bias was detected by Begg's test, the *P-value*s was 0.348. Therefore, we can assume there were insignificant in publication bias.

## DISCUSSION

Although little is know about the correlation of Se for HCC, several studies have shown that there is an inverse correlation between HCC risk and Se level. We performed the meta-analysis with the latest resouce on the correlation between HCC risk and Se level. Combining the results from 9 studies, which applied individual levels of Se measured in serum, blood or toenails, indicated that there was a significant high risk of HCC correlate with low levels of Se (Figure 2). As can be seen in Table 2, the pools OR for Se level in HCC patients in all studies was −1.08 (95% CI = −0.136, −0.8) compare with the healthy controls. In the subgroup analysis by study design, race, sample collection, we found these factors significantly influnce the role of low Se level in incidence of HCC. Additionally, significant heterogeneity was found in subgroup analyses and might detract from the validity of the results. Thus, more research is needed to investigate the correlations between HCC risk and low Se level in Asian and the studies with blood collection.

**Table 2:** Results of this meta-analysis

Association					Heterogeneity	
	Num	SMD (95% CI)	*P* value	Model	I^2^ (%)	*P* value
overall	9	−1.08 (−0.136,– 0.08)	*P* < 0.001	R	74.3	*P* < 0.001
Study design							
case-control	6	−1.388 (−1.751, −1.026)	*P* < 0.001	R	47.6	*P* = 0.09
cohort	3	−0.733 (−0.945, −0.520)	*P* < 0.001	R	52.5	*P* = 0.122
Race							
Caucasian	3	−0.918 (−1.443, −0.393)	*P* < 0.001	R	50.5	*P* = 0.133
Asian	6	−1.115 (−1.526, −0.784)	*P* < 0.001	R	80.6	*P* < 0.001
Sample collection							
Serum	3	−1.113 (−1.472, −0.755)	*P* < 0.001	R	23.5	*P* = 0.27
Blood	5	−1.241 (−1.795, −0.678)	*P* < 0.001	R	84.7	*P* < 0.001
Toenail	1	−0.889 (−1.081, −0.697)	*P* < 0.001	R	0	*P* < 0.001

Num, number; SMD, standardized mean difference; CI, confidence intercal; R, randomeffects model.

Evidence indicated several mechanisms for Se antitumous effect. Actually, antioxidant properties of selenoproteins are relevant in protection from cancers [25]. Evidence from animal models and primary human hepatocytes implicate Se in liver cancer development [1–4], whereas progressive cancer grade were correlated with decreasing Se concentrations in HCC tumor tissues [26]. Nataliya stated that in the case of HCC patients with low Se levels, Se supplementation could be considered for chemoprevention [2]. It is also showed a 50% Se-induced reduction in HCC occurrence in China [27].

As far as we know, this is the first systematic review to investigate the correlation between Se levels with HCC in patients. However, the limitations of the present study must be considered. The reslut of the present study found a significant heterogeneity. Study design subgroup showed I^2^ value was decreased. The result suggested that the major source of the heterogeneity might be the study design. For the age subgroup analysis, the age groups did not match perfectly. We have use an age range of 20–60 years as “adult” and an range of older than 60 years as “older”. However, most of the studies with ages ranging between 20–80 years in HCC patients. Thus, our finding is unlikely to be the result of unequal age ditribution. We were not able to investigate the effect of sex subgroups because of a lack of data. Additionally, Begg's tests results showed that there was no significant publication bias in this meta-analysis.

In conclusion, this meta-analysis supports an inverse correlation between Se level and the risk of HCC in humans. However, both epidemiological survey and biological research should be further conducted to illustrate and validate whether Se supplement is beneficial for prevention and treatment of HCC. The exact mechanism needs to be further investigated.

## MATERIALS AND METHODS

### Search strategy

Studies were identified by searching PubMed, EMBASE, web of science, Cochrane Library, Springer Link, Chinese National Knowledge Infrastructure (CNKI), and Chinese Biology Medicine (CBM) before August 2016, using the following Mesh terms: (“Liver Neoplasms” [MeSH] or “liver cancer” or “Hepatocellular Cancer” or “Hepatic Neoplasm”) and (“selenium”or “Se”). Reference lists of all eligible studies were screened to identify potentially eligible studies. Emails were sent to the authors of identified studies for additional information if necessary.

### Selection criteria

Studies included in this meta-analysis have to meet the following criteria: (1) human study; (2) case-control study or cohort study studying on correlation between Se and HCC; (3) all patients with the diagnosis of HCC confirmed by pathological or histological examination; (4) studies providing serum levels of Se for both subjects with HCC and healthy controls; (5) subjects with no other diseases and no drugs intake which might influence the levels of Se. Studies were excluded when they were: (1) *in vitro* or laboratory study; (2) animal study; (3) not case-control study or cohort study; (4) based on incomeplete data; (5) review or case report.

### Data extraction and assessment of study quality

Data were independently extracted by two reviewers using a standardized data extraction form. Discrepancies were resolved by discussion and if consensus was not achieved the decision was made by all the reviewers. The following data was extracted from every article: first author, year of publication, study design, race, age and sex, sample size, years of follow-up, levels of Se and sample collection.

### Statistical analyses

Statistical analysis was conducted by using STATA version 12. The correlation of Se level and HCC was estimated by SMD with 95% CI. Both fixed and random effects models were assessed, but the latter was preferentially used when heterogeneity was detected. The I^2^ statistic was used to determine the level of heterogeneity potential sources of heterogeneity were explored using subgroup analysis to check the influence of the following determinants: study design, race and sample collection. Publication bias was explored Begg's Test. *P value* less than 0.05 was considered statistically significant.
